# Effects of Warm Ischemic Time on Gene Expression Profiling in Colorectal Cancer Tissues and Normal Mucosa

**DOI:** 10.1371/journal.pone.0053406

**Published:** 2013-01-07

**Authors:** Valeria Musella, Paolo Verderio, James Francis Reid, Sara Pizzamiglio, Manuela Gariboldi, Maurizio Callari, Milione Massimo, Loris De Cecco, Silvia Veneroni, Marco Alessandro Pierotti, Maria Grazia Daidone

**Affiliations:** 1 Department of Experimental Oncology and Molecular Medicine, Fondazione IRCCS Istituto Nazionale dei Tumori, Milan, Italy; 2 Unit of Medical Statistics and Biometry, Fondazione IRCCS Istituto Nazionale dei Tumori, Milan, Italy; 3 Molecular Genetics of Cancer, Fondazione Istituto FIRC di Oncologia Molecolare, Milano, Italy; 4 Department of Pathology, Fondazione IRCCS Istituto Nazionale dei Tumori, Milan, Italy; 5 Scientific Directorate, Fondazione IRCCS Istituto Nazionale dei Tumori, Milan, Italy; Macquarie University, Australia

## Abstract

**Background:**

Genome-wide gene expression analyses of tumors are a powerful tool to identify gene signatures associated with biologically and clinically relevant characteristics and for several tumor types are under clinical validation by prospective trials. However, handling and processing of clinical specimens may significantly affect the molecular data obtained from their analysis. We studied the effects of tissue handling time on gene expression in human normal and tumor colon tissues undergoing routine surgical procedures.

**Methods:**

RNA extracted from specimens of 15 patients at four time points (for a total of 180 samples) after surgery was analyzed for gene expression on high-density oligonucleotide microarrays. A mixed-effects model was used to identify probes with different expression means across the four different time points. The p-values of the model were adjusted with the Bonferroni method.

**Results:**

Thirty-two probe sets associated with tissue handling time in the tumor specimens, and thirty-one in the normal tissues, were identified. Most genes exhibited moderate changes in expression over the time points analyzed; however four of them were oncogenes, and two confirmed the effect of tissue handling by independent validation.

**Conclusions:**

Our results suggest that a critical time point for tissue handling in colon seems to be 60 minutes at room temperature. Although the number of time-dependent genes we identified was low, the three genes that already showed changes at this time point in tumor samples were all oncogenes, hence recommending standardization of tissue-handling protocols and effort to reduce the time from specimen removal to snap freezing accounting for warm ischemia in this tumor type.

## Introduction

With the introduction of new genomic technologies such as tissue-based RNA microarrays, patterns of gene expression identified by microarray analyses have been discovered that stratify tumors and predict the clinical outcomes in different cancer types [1]. Some of them have been successfully used for identifying patients that can benefit of specific treatment and FDA (Food and Drug Administration) -approved tests based on breast cancer gene signatures are now commercially available. As a consequence, collecting in tissue banks surgical specimens that can be used for these analyses has become a mandatory issue for understanding the correlative results of the majority of current clinical trials. However, the variability in tissue handling and processing of surgical specimens may affect the reproducibility and interpretation of results. Several variables, including tissue manipulation, warm ex-vivo ischemia and storage times can potentially alter mRNA expression levels and adversely affect the validity of studies that used clinical specimens [2]. All these variables need to be carefully investigated in order to set up guidelines for tissue banking [3]. The organization of qualified and certified biobanks should be the basis to guarantee networking of activities and availability of quality-certified biological material.

The purpose of this pilot study was to explore the effects of tissue handling time in precisely documented tissue samples that followed the routine processing standards in our Institution (Fondazione IRCCS Istituto Nazionale dei Tumori, INT-MI), for developing a clinically applicable method of sampling tumors in tissue banks that can be safely used for microarray analyses. We used the colorectal cancer (CRC) model. CRC is the second most frequent cause of cancer death in Western countries and despite significant advances in its management, the overall survival for advanced and metastatic disease has changed little over the last 20 years, with five years at almost 90% for early and 15% for late tumors [4]. As a consequence, there is a pressing need for new biomarkers to improve the detection and the clinical treatment of CRC. Using high-density oligonucleotide microarrays we investigated sequential effects of tissue handling in specimens obtained from 15 CRCs and in their matched normal tissue collected at our Institution and left at room temperature at different time points after surgery. The primary study outcome was to evaluate the effect of the time on tumor samples and possibly select specific genes whose expression is time-related, that could be used as detectors of tissue degradation. Additionally, to identify genes influenced by time irrespectively of the sample type (normal or tumor), we also investigated the time effect in normal tissues and compared the differential expression between the two tissue types accounting for time.

Our results show that the impact of tissue handling time on the whole gene expression profile could be considered minor in colon and that a reasonable threshold for collecting specimens could be 60 minutes after surgery, when no gene alterations were observed. Such samples may be used to generate reproducible microarray profiles to aid treatment decision making utilizing clinicogenomic models.

## Materials and Methods

### 1 Ethics Statement

All patients whose biological samples were included in the study signed an informed consent, approved by the Independent Ethical Committee of the INT-MI, to donate to INT-MI the leftover tissue specimens after completing diagnostic procedures for research purposes. The Independent Ethical Committee of INT-MI approved the use of the samples for this specific study in the framework of a project in biobanking quality control.

### 2 Study Design and Sample Handling

The tumor and the normal counter-part samples used in the experiments were prospectively collected from 15 patients who underwent surgical resection at the INT-MI and whose tumors were representative of the different pathologic stages of this tumor type. At the histological routinely examination all tumor specimens were classified moderately differentiated colonic Adenocarcinomas NOS (grade G2 according to the American Joint Committee on Cancer 2010 http://www.cancerstaging.org/). The clinico-pathologic and histological details are listed in Table 1. Six fragments from each sample were obtained and were randomly left at room temperature at different time points as follows: three fragments at <20 min. (T0), one fragment at 60 min. (T1), one fragment at 180 min. (T2) and one fragment at 360 min. (T3). Time was measured starting from patient’s surgical excision and the first timepoint analysed (T0) was processed and frozen within 20 min. from surgery. Samples were transported from theater to pathology at room temperature without ice. The total number of analyzed fragments was 180 (90 normal samples and 90 tumor samples). Neoplastic samples were obtained from the central area of the neoplasia, avoiding selecting necrotic material or transition zones with healthy mucosa. Samples of colonic healthy mucosa were resected at least 20 centimeters far from the neoplasia and distant from the surgical resection margins. The control was costituited exclusively by normal mucosa, stripped from the luminal surface.

**Table 1:** Characteristics of the cases sampled for RNA isolation.

Case	Gender	Age	Tumor location	Grade	Stage	Lymphonodes
1	F	80	sigma-rectum	G2	pT2	neg
2	F	45	sigma-rectum	G2	pT2	neg
3	M	47	sigma	G2	pT2	neg
4	F	72	colon	G2	pTis	neg
5	M	67	sigma-rectum	G2	pT1	neg
6	M	35	colon	G2	pT2	neg
7	M	70	sigma-rectum	G3	pT2	neg
8	F	76	sigma-rectum	G2	pT4	pos
9	F	60	sigma	G2	pT3	pos
10	M	73	sigma-rectum	G2	pT2	neg
11	F	54	sigma-rectum	G2	pT3	pos
12	M	32	sigma-rectum	G3	pT3	pos
13	F	73	ileum	G2	pT2	neg
14	M	55	sigma-rectum	G2	pT4	pos
15	M	43	sigma	G2	pT4	pos

### 3 RNA Extraction and Evaluation

Tissue samples were stored at −80°C until RNA extraction. Total RNA was extracted from 10–20 mg of tumor samples and from 30–40 mg of normal samples. Tissues were mechanically disrupted and simultaneously homogenized in the presence of QIAzol Lysis reagent (Qiagen, Valencia, CA, USA), using a Mikrodismembrator (Braun Biotech International, Melsungen, Germany). RNA was extracted using the miRNeasy Mini kit (Qiagen) according to manufacturer’s instructions. Purification and DNase digestion were performed using two different kits: RNeasy MinElute Cleanup (Qiagen) was employed for up to 45 *µ*g of RNA while RNeasy Mini kit (Qiagen) was used for RNA ranging between 45 and 100 *µ*g. RNA concentrations were measured with the NanoDrop ND-100 Spectrophotometer (NanoDrop Technologies, Wilmington, DE) while RNA quality was assessed with the Agilent 2100 Bioanalyzer (Agilent Technologies, Palo Alto, CA) using the RNA 6000 Nano kit (Agilent Technologies). The RNA Integrity Number (RIN) [5] was determined using the software provided by the manufacturer.

### 4 Gene Expression Profiling

RNA samples were processed for microarray hybridization by the Functional Genomics core facility at INT-MI. Briefly, 800 ng of total RNA was reverse transcribed, labeled with biotin and amplified overnight (14 hours) using the Illumina RNA TotalPrep Amplification kit (Ambion, Austin, Texas, USA) according to manufacturer’s protocol. One ug of the biotinylated cRNA sample were mixed with the Hyb E1 hybridizatioin buffer containing 37.5% (w/w) formamide and then hybridized to Sentrix Bead Chip HumanHT12_v3 (Illumina, Inc., San Diego, CA) at 58°C overnight (18 hours). The array represents over 48000 bead types, each with a unique sequence derived from human genes in the National Centre for Biotechnology Information Reference Sequence and UniGene database. Array chips were washed with manufacturer’s E1BC solution, stained with 1 ug/ml Cy3-streptavidine (Amersham Biosciences; GE Healthcare, Piscataway, NJ, USA) and eventually scanned with Illumina BeadArray Reader.

### 5 Real Time PCR

Taqman® gene assays were used for validation of ABL1 (Hs00245443), FOSB (Hs01547109), JUN (Hs00277190) genes in the Tumor samples and HIST1H1D (Hs00271187), HIST1H1E (Hs00271195), HIST1H4E (Hs003743461), HIST4H4 (Hs00545522) in both Tumor and Normal samples. All genes were normalized to 18S (HS03003631), while the three Tumor-associated genes were also normalized to ACTB (HS03023942) and GAPDH (Hs00266705). Briefly, cDNA was synthesized in duplicate for the validation of ABL1, FOSB and JUN and in a single reaction for the validation of histone genes from 500 ng of total RNA using the High-Capacity cDNA Reverse Transcription Kit (Applied Biosystems, Foster City, CA) according to the manufacturer’s instructions. Real-Time qPCR was performed using the FAST chemistry (Applied Biosystems) with the gene-specific assays in ABI PRISM 7900 HT Real-Time PCR system (Applied Biosystems) using 10 ng of cDNA.

### 6 Data Analysis

#### 6.1 Microrarray data pre-processing

Raw data were obtained from scanned images using the Illumina BeadStudio software (version 3.3.8) and pre-processed using the lumi package [6] of the Bioconductor project [7]. The signal mean, the detection rate and the between array distances were evaluated in the quality control step. Two of the 90 profiles from tumor samples and two from the 90 profiles from normal samples did not pass quality controls and were discarded in subsequent analysis. Data were normalized using the Robust Spline Normalization method and probes with a detection p-value <0.01 in less than 10% of samples were filtered out. All microarray data are MIAME compliant and the raw data were deposited into the NCBI’s Gene Expression Omnibus (GEO) database (http://www.ncbi.nmlm.nih.gov/projects/geo/) with accession number GSE37182.The association between baseline gene profiles and clinic-pathological features was analyzed by One-way ANOVA.

#### 6.2 Time course analyses

In order to identify probes in tumor samples differently expressed across the four different time points (*time course expression analysis*) a mixed-effects model was implemented on the microarray data by considering the factor *Time* (T0, T1, T2, T3) as fixed and the factor *Patient* as random [8]. The model was implemented by considering as dependent variables the log (base 2) expression value of each probe. The p-values of the model were adjusted with the Bonferroni method [9]. For the validation study the same approach was applied by considering as dependent variables the –ΔCt values obtained from qPCR performed on the genes of interest. Statistical analysis was performed implementing a specific code developed in SAS ® software v. 9.2 (SAS Institute Inc. Cary, NC). Superimposable results were produced by using the programming language R v. 2.12.0 [10] as well as by using the freely distributed and open-source EDGE software package [11]. An identical statistical procedure was subsequently applied in processing the data from Normal tissue. RIN values corresponding to both normal and tumor samples were analyzed following the same approach used for microarray data by also considering the factor *Type* (normal or tumor) as fixed (*time course RIN analysis*).

#### 6.3 Gene set analysis

Gene set functional analysis using the Gene Ontology (2010) [12]; [13] databases was performed using the Bioconductor packages GOstats [14] (v. 2.16.0) and Category (v. 2.16.0) with default parameters (over-representation with p-value <0.01).

## Results

### 1 RIN Analysis

**Figure 1:**
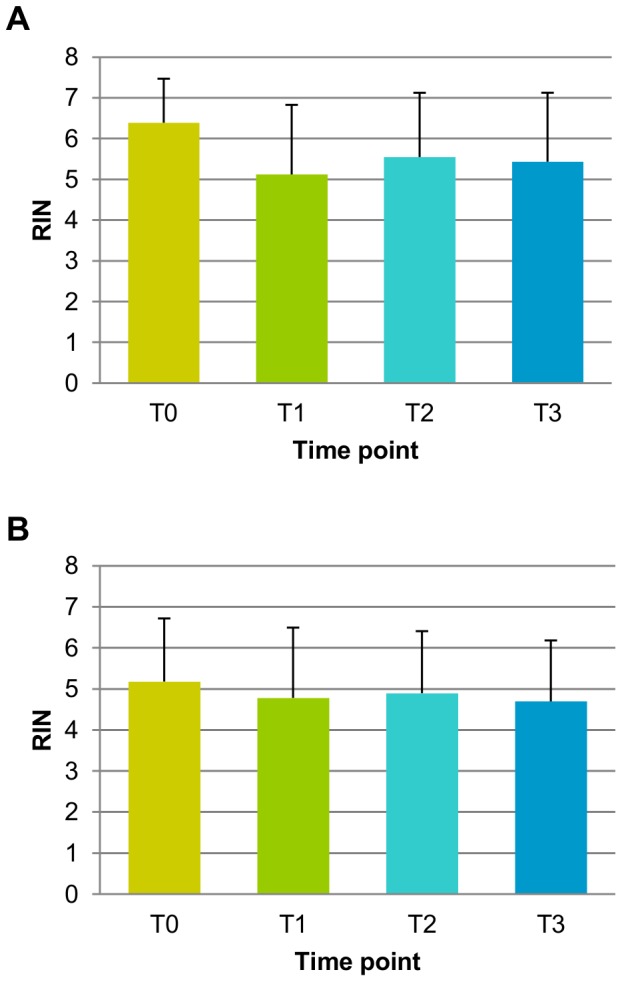
RIN analysis. RIN distributions at each time point for both tumor (A) and normal (B) samples. A higher RIN indicate a higher RNA quality.

The RNA Integrity Number (RIN) of all samples over all time points had a median value of 5.8 with an inter-quantile range of 1.95. Tumor samples had on average a higher RIN value than the normal samples (median of 6.4 and 5.35 respectively), especially at the first time point T0. Similar distributions of RIN values could be observed across the different time points and considerable variation was observed for each sample within the first time point T0 (RIN variance median = 1.668, IQR = 2.084) which was more pronounced in the normal samples. The time course RIN analysis showed that RIN values had a tendency to decrease over time (overall p-value for Time = 0.0565 and for single contrasts compared to T0, T1 = 0.0214, T2 = 0.1705 and T3 = 0.0529). The distribution of RIN values differ significantly (p-value = 0.0008) in Tumor samples with respect to the Normal ones (Fig. 1).

By considering all the four time points we identified a subset of 6 normal and 7 tumor cases the RIN values of which was ever higher than five and time course expression analysis was performed also for this subset of samples. It should be stressed that no association was observed between gene profiles on tumor samples at T0 and stage (p-value = 0.59) or location (p-value = 0.95) of the disease.

### 2 Time Course Expression Analysis

After the quality control of microarray data, the expression profiles of 4 samples (2 profiles from tumor and 2 from normal samples) were removed. These outliers belonged to different time points of two patients in the case of normal samples and to the same patient for tumor samples. Due to the nature of the study design it was crucial to have for each patients a full pictures across all the time points and thus all profiles associated with those patients were not take into consideration in the time course analysis. As a consequence, a total of 13 patients were used in the Normal dataset which contained 17895 probes and 14 in the Tumor dataset containing 18007 probes. Both datasets shared 16698 common probes.

The probes identified by the stringent Bonferroni correction (p-value <0.05) method in the *Time* factor or in at least one of the time contrasts (T0 baseline) in the Normal and Tumor datasets are listed in Table 2 (N = 32) and Table 3 (N = 31) respectively. In these tables, the rows have been ordered according to the raw p-values deriving from the *Time* factor and a star (*) under the columns ‘Time’, ‘T1’, ‘T2’, or ‘T3’ indicates from which contrast the probe was selected. Heatmaps in the panel A and B of Figure S1 graphically represent the direction of the expression alteration at each time point in the Tumor and Normal datasets, respectively.

**Table 2:** Top candidates identified in the Normal dataset (Bonferroni P<0.05).

ProbeID	ILMN	Symbol	P-value	Time	T1	T2	T3
1030017	ILMN_1746435	HIST1H1E	2.32e-12	*		*	*
5080246	ILMN_1779373	HIST1H2BF	6.50e-12	*		*	*
2230619	ILMN_1681542	HIST1H4E	9.69e-12	*		*	*
1440747	ILMN_2165369	HIST1H4B	2.43e-11	*		*	*
1300504	ILMN_2188451	HIST1H2AH	3.10e-11	*		*	*
4180360	ILMN_2053992	HIST4H4	4.02e-11	*			*
2680450	ILMN_1749789	HIST1H1D	1.14e-10	*			*
450753	ILMN_1680937	HIST1H2BC	1.90e-10	*		*	*
430546	ILMN_1716195	HIST1H2BG	1.95e-10	*		*	*
5960086	ILMN_1901419		5.25e-10	*		*	*
7380162	ILMN_1694699	HIST1H2AK	2.29e-09	*			*
4640082	ILMN_2057836	RNU2-1	2.93e-09	*			*
5270731	ILMN_1722059	SAFB	4.08e-09	*			*
2000133	ILMN_1768139	RNU12	1.07e-08	*			*
4570725	ILMN_1664706	LOC653604	1.38e-08	*			*
2030678	ILMN_1747589	HIST2H2AB	3.14e-08	*			*
990176	ILMN_1739423	RN7SK	3.89e-08	*			*
460386	ILMN_1759954	PTMA	7.93e-08	*			*
2230215	ILMN_1724341	CXorf45	1.50e-07	*			*
1500674	ILMN_1666179	HIST2H3C	1.63e-07	*			*
6290612	ILMN_2413331	TMEM107	1.91e-07	*			
6770438	ILMN_1653251	HIST1H1B	2.31e-07	*			*
6520424	ILMN_1712184	HIST1H3C	2.67e-07	*			*
6590594	ILMN_1792689	HIST1H2AC	5.44e-07	*			
510441	ILMN_1726815	HIST1H3G	6.14e-07	*			
3870678	ILMN_1756849	HIST1H2AE	8.47e-07	*			
650192	ILMN_1736820	HIST1H2BM	1.10e-06	*			
2140524	ILMN_1721127	HIST1H3D	1.14e-06	*			
2970019	ILMN_1751120	HIST1H4H	1.52e-06	*			*
2690561	ILMN_1689725	RPLP1	1.61e-06	*			
1300167	ILMN_1737170	FLII	4.18e-06				*
4890279	ILMN_2115340	HIST2H4A	1.71e-05				*

**Table 3:** Top candidates identified in the Tumor dataset (Bonferroni P<0.05).

ProbeID	ILMN	Symbol	P-value	Time	T1	T2	T3
4590440	ILMN_1708922	ABL1	4.00e-11	*		*	*
6380717	ILMN_1789074	HSPA1A	8.57e-10	*			*
1340600	ILMN_1659936	PPP1R15A	1.14e-09	*			*
1030017	ILMN_1746435	HIST1H1E	1.73e-09	*			*
7160239	ILMN_1751607	FOSB	1.98e-09	*		*	*
6510367	ILMN_1806023	JUN	7.87e-09	*		*	*
2260309	ILMN_1704056	RPPH1	9.68e-09	*			*
4920110	ILMN_1718977	GADD45B	1.20e-08	*			*
4490520	ILMN_1798706	EBI2	2.21e-08	*			*
2230619	ILMN_1681542	HIST1H4E	2.75e-08	*			*
2680450	ILMN_1749789	HIST1H1D	3.02e-08	*			*
4180360	ILMN_2053992	HIST4H4	2.56e-07	*			*
4280113	ILMN_1773154	NFKBIA	3.16e-07	*			*
5960086	ILMN_1901419		4.18e-07	*			*
870338	ILMN_1762899	EGR1	6.31e-07	*			
5890739	ILMN_1739985	TAGAP	1.18e-06	*			*
60470	ILMN_1720771	STX11	1.40e-06	*			*
6860377	ILMN_1781285	DUSP1	1.47e-06	*			*
3800168	ILMN_1775708	SLC2A3	2.07e-06	*			*
830619	ILMN_1676984	DDIT3	2.25e-06	*			*
3400332	ILMN_1802205	RHOB	2.54e-06	*			
650241	ILMN_1721833	IER5	2.58e-06	*			
7570411	ILMN_1761314	NFS1	2.87e-06				*
5050162	ILMN_1780582	CD83	2.92e-06				*
6020470	ILMN_1756937	ST8SIA4	4.26e-06				*
1940047	ILMN_1703538	AIF1	4.36e-06				*
7550484	ILMN_1760347	SRGN	4.93e-06				*
4490176	ILMN_1656011	RGS1	9.37e-06				*
5700670	ILMN_1668417	WASPIP	9.49e-06				*
3170128	ILMN_2353732	CD8A	1.04e-05				*
620717	ILMN_1773352	CCL5	1.08e-05				*

Five probes (genes) exhibiting different means of expression across the four time points were identified both in the Normal and the Tumor datasets (*HIST1H1D, HIST1H1E, HIST1H4E, HIST4H4 and 5960086).* The majority of the variability observed derives from the last time point T3 at 360 minutes when comparing to the baseline T0. This is especially true in the Tumor dataset whereas some probes deriving from the T2 contrast emerge in the Normal dataset. All genes identified in the Tumor dataset are consistently up-regulated over time; the same applies to the Normal dataset with a few exceptions. On the whole however, most genes do not exhibit drastic changes in levels of expression. Functional annotation of the genes from the two lists (using both Gene Ontology and KEGG pathways databases) highlighted that 22 of the 32 genes modulated in normal tissue were histones, involved in nucleasome organization and chromatin assembly. Four of them were also time-dependent in the tumor samples, together with other 27 genes with various functions involved in cancer, including oncogenes, such as *JUN*, *FOSB*, *ABL1* and *EGR1*. Another gene commonly modulated in Normal and Tumor tissues was *RNU11*, a small nuclear RNA.

An identical analysis was performed using the subset of samples with a RIN higher than five which identified only 6 probes in the Normal dataset and 6 in the Tumor dataset, dataset probably because of the reduced sample size. No common probes where found in the two lists. Five of 6 probes (*HIST1H1E*, *HIST1H4B*, *HIST1H4E*, *HIST4H4*, *5960086*) in the Normal dataset were also present in the analysis using all samples and 2 (*HSPA1A*, *IER5*) were in common in Tumor dataset.

### 3 RT-PCR Validation

**Figure 2:**
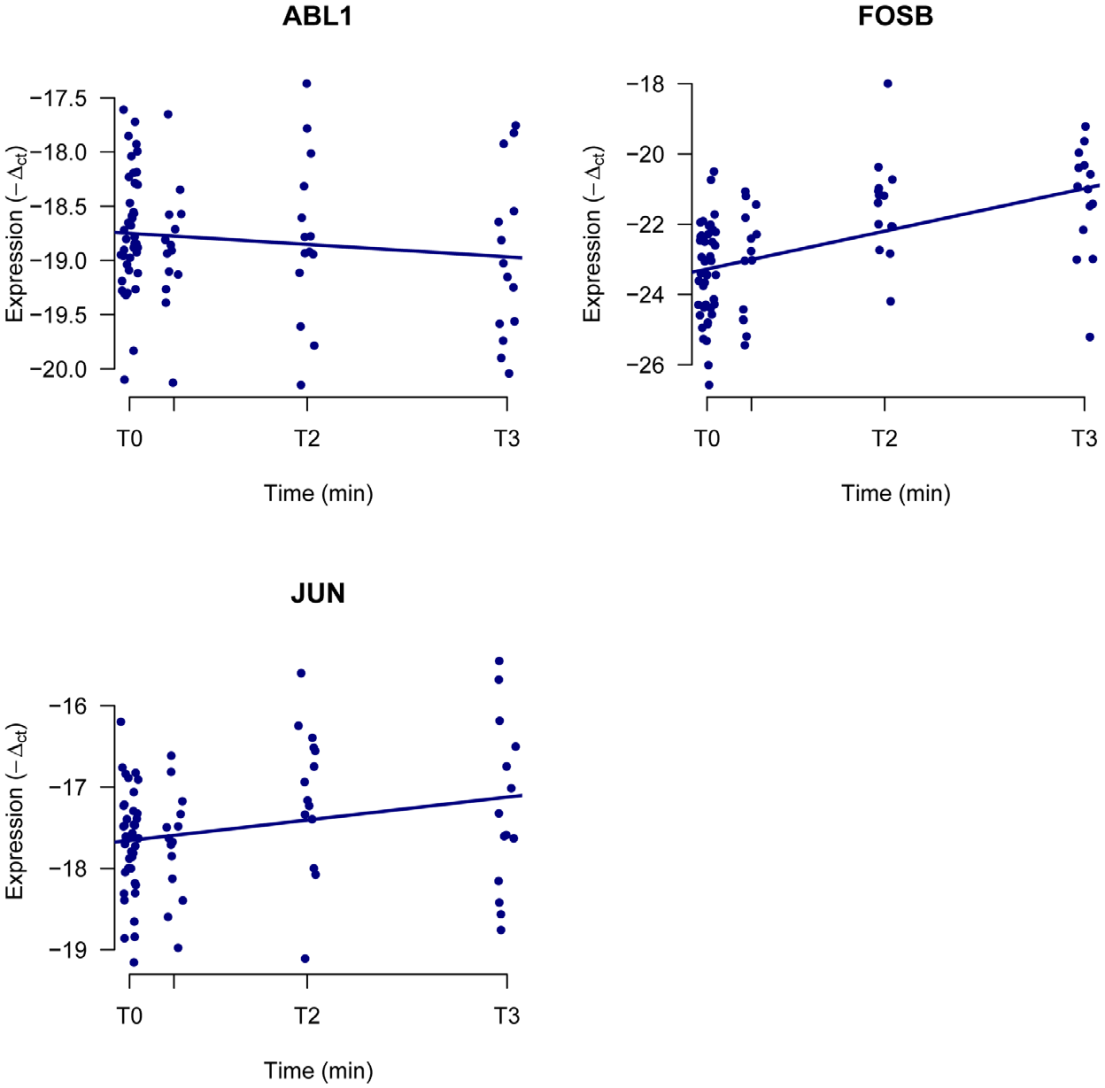
Time course for selected genes. RT-PCR values of FOSB, JUN and ABL1 (−ΔCt) normalized to the house-keeping gene 18S.

The three genes *that were significant* for ‘Time’, ‘T2’ and the ‘T3’ contrasts *in the Tumor samples (JUN*, *FOSB* and *ABL1)* were selected for technical validation with Real Time PCR (RT-PCR) analysis in the same Tumor tissues belonging to the 14 different patients analyzed by microarrays. Two of them (*JUN and FOSB)* confirmed the results from the arrays. Figure 2 reports the expression dynamic over time of the genes, normalized to 18S. Normalization using GAPDH and ACTB housekeeping genes confirmed these results (data not shown). ABL1 was not validated as ischemia associated gene likely due to the weak level of association between microarray and RT-PCR data. This can be partially explained by the limited expression variability of this gene in our samples compared with JUN and FOSB (Fig. S2). Four genes commonly modulated in normal and tumor tissue, were also analyzed (HIST1H1D, HIST1H1E, HIST1H4E and HIST4H4); only one of them, HIST1H4E, confirmed the microarray data.

## Discussion

We studied the effect of tissue handling time on global gene expression using CRC specimens sub-sampled and snap-frozen at different time points post-surgery, following routine tissue handling protocols of a Pathology Unit. RNA quality assessed by the RNA Integrity Number (RIN) showed a slight trend of decrease associated to time with tumor samples exhibiting higher and less variable RIN values at T0 compared to the normal samples. The presence of degradation at T0 could indicate the variability of the procedure or a specific sensitivity of RNA from colon tissue. Results obtained for tumor samples are in agreement with what found by Bray et al. [15]. All the samples were used for microarray analysis irrespective of their RIN number to understand if the microarray platform could highlight better proxies of quality for tissue handling. The distribution of feature intensities per array was highly similar across all the 180 arrays analyzed, suggesting that all RNAs performed at the same level and that hybridizations were done similarly; only 4 samples did not pass the quality controls.

To assess the variability of gene expression measurements as a function of time, each feature (probe) was modeled respect to the Time categories and adjusted for the Patient factor. This was done separately in both Tumor and Normal datasets. Using a Bonferroni correction of P<0.05, thirty-one probes were identified as time-dependent in the tumor specimens and thirty-two in the normal samples. Many of the genes identified in the tumor dataset were DNA-binding proteins involved in different processes: some corresponded to genes involved in cancer, such as *ABL1*, *JUN*, *FOSB*, *NFKB1A* and *CCL5*; four probes belonged to histones (*HIST1H1E*, *HIST1H4E*, *HIST1H1D* and *HIST4H4*), others were transcription factors (*DDIT3*) or genes involved in transcription such as *EGR1* and *PPP1R15A*. Six genes were involved in apoptosis, one of the processes induced by tissue resection. Most of the genes identified (N = 22) were significant in the Time comparison involving all time points, 16 were identified in T3 versus T0 contrast and only 3 genes (*ABL1*, *JUN* and *FOS*) at both time points T2 and T3. Nine genes changed only at T3. On the other hand, many of the genes identified in the normal samples (N = 21) corresponded to genes coding for Histone proteins, and three were small nuclear RNAs (*RNU2-1*, *RNU11* and *RNU12*). Thirty-two genes changed over all the four time points analyzed; 17 of them were significantly different at time point T3 and 8 at both time points T2 and T3. Two genes changed only at T3. By performing the same analysis only using the samples with a RIN above five very few genes were identified (6 probes in both datasets) of which mainly histones were in common with the previously identified genes. The results from these two approaches indicate that the changes observed over time are fairly negligible. Indeed, when we compared the tissue types between both Normal and Tumor datasets a very large number of differences where observed, as expected, irrespective of the quality of the RIN. Further, functional analysis of these probes using the Kyoto Encyclopedia of Genes and Genomes (KEGG) pathway database showed that these differences matched with eleven significant pathways involved in cancer progression including Cell cycle, DNA replication and the p53 signaling pathway (data not shown).

The changes observed in the genes for both normal and tumor tissues mainly occurred at T3, although some genes were already altered at T2, suggesting that a critical time point lays at 60 minutes at room temperature. Interestingly, the three genes that showed changes already at T2 in tumor samples were all oncogenes, which suggests to carefully consider alterations of these genes while handling tumor samples and to use a more conservative time threshold (i.e. 60 minutes) for sample collection. The expression levels over time of most of the genes were increased. The present finding is in agreement with what found by Dumur et al. [16] on ovary cancer cases and by Bray et al. [15], in CRC, where the number of probes with increased expression augmented with time. As discussed by the authors of the two studies, this is opposite to what expected since handling time should favor degradation of RNA transcripts and may in part at least reflect an active modulation of gene expression. Comparison between GO classification of differentially expressed genes in our (FDR adjusted p-values<0.05) and Bray’s analyses showed few common enriched terms (‘protein dimerization activity’ and ‘transcription factor activity’). Possible explanations for these results could be the different time points evaluated (up to 360 minutes in our study and up to 120 minutes in Bray’s study), different procedures followed for sample collection (surgical specimens that followed the routine processing standard vs tumor biopsies) and different microarray platforms used for expression analyses (Illumina vs Affymetrix).

Two of the three genes selected for the validation *in tumor tissues* (*JUN and FOSB) confirmed the array results*. These genes were also among the mRNAs most significantly affected by time to freezing in a breast cancer study [17]. As reported by these authors, JUN and FOSB are stress induced immediate early transcription factors which are components of AP-1 dimers, and these dimers have been found altered by ischemia in different tissues including prostate and colon cancer. In ischemic and riperfused cells the two genes induce proliferation and apoptosis and could be closely related to attemps for degradation or regeneration of injuried tissues [18]. Five probes were shared among normal and tumor specimens. They identified genes from the histone family (*HIST1H1D*, *HIST1H1E*, *HIST1H4E* and *HIST4H4*) involved in DNA organization, and a small nuclear RNA (*RNU11*). Only one of these genes (*HIST1H4E*) validated the microarray data. The high sequence similarity of the histones could explain the high number of modulated histones identified, that could be due to partially aspecific hybridization and could be the reason for the lack of validation with an independent technique.

In this paper we have described for the first time the effect over time on handling up to 6 hours of normal colorectal tissue, which is frequently used as control in genome wide analyses. Interestingly, normal tissue showed less degradation than its corresponding tumor specimen, both in terms of RNA quality (RIN value) and of modulated genes that were mainly histones. Our results favor the storage of normal tissue, in addition to the tumoral.

The overall changes in gene expression seen in the specimens analyzed in the current study, where more than 16000 probes were investigated, do not seem to correlate with a global transcriptome event that one might expect, at least during the time frame analyzed in this study (<20 minutes, 60 minutes, 180 minutes and 360 minutes). Considering the very low number of affected genes found, the impact of tissue handling time on the overall gene expression profiling, at least in CRC, could be considered minor and would not be expected to play a major role in gene expression-based tumor stratification.

Our results agree with that of Dumor et al. [16] and of Micke et al. [19] that showed no relevant changes in ovary cancer and with what Hatzis et al. found in breast cancer [20]. Other studies have shown significant alterations in RNA after 30 minutes of tissue extirpation[15]; [21]; [22]. Since our design did not include early times, we cannot exclude that relevant changes have already occurred before our first time point (20 minutes). However, the aforementioned studies typically have very low sample sizes and would need further validation.

Significant alteration of gene expression profile were observed in our previous study on breast cancer specimens where tissue handling time altered the expression of genes included in the commonest breast cancer predictive gene signatures [23]. These data were in agreement with Borgan et al. [17], that found miRNA (microRNA) and mRNA expression alterated in breast cancer with ischemia time up to six hours. Indeed, Hatzis and colleagues [20] in breast cancer showed no relevant changes in expression levels of single genes and multigene signatures, probably because they considered 40 minutes as longest time point at room temperature or up to 180 minutes but using the RNA-later to preserve RNA integrity.

Despite partial disagreement on its extent, time of tissue handling can have an effect on gene expression, and probably might vary with tissue and with tumor type, including those that could exhibit greater sensitivity to hypoxia effects, such as brain tumors [24].

### Conclusions

The findings that four of the few genes significantly different among the timepoints analyzed in the Tumor samples were oncogenes, hints that analysis of their expression in tumor specimens could lead to misleading results. Even if tissue handling time has a weak impact on the overall gene expression profiling, the deregulation of genes directly involved in tumor processes implies that tissues should be stored at early times after surgery (in our context no more than one hour) and strongly support its introduction as guideline for tissue repositories.

## Supporting Information

Figure S1 **Expression alteration at each time point in the Tumor (A) and Normal (B) datasets**
(PPTX)

Figure S2 **Scatter plots of RT-PCR ΔCt values (x-axis) versus log2 microarray intensity values(y-axes) for ABL1, JUN and FOSB genes.** In each graph the Pearson correlation coefficient (R) was used to measure the strength of association.
(PPTX)
