# Correction: Effects of Warm Ischemic Time on Gene Expression Profiling in Colorectal Cancer Tissues and Normal Mucosa

**DOI:** 10.1371/annotation/11071e0f-4f09-4b1f-93ec-f1362610ce33

**Published:** 2013-06-07

**Authors:** Valeria Musella, Paolo Verderio, James Francis Reid, Sara Pizzamiglio, Manuela Gariboldi, Maurizio Callari, Massimo Milione, Loris De Cecco, Silvia Veneroni, Marco Alessandro Pierotti, Maria Grazia Daidone

The seventh author's name was spelled incorrectly. The correct name is: Massimo Milione

