# Supplementary figures and images for: Effects of Warm Ischemic Time on Gene Expression Profiling in Colorectal Cancer Tissues and Normal Mucosa
Source: PLoS One. 2013 Jan 7;8(1):e53406. doi: 10.1371/journal.pone.0053406 (PMC3538764; doi:10.1371/journal.pone.0053406)

## Slide 1
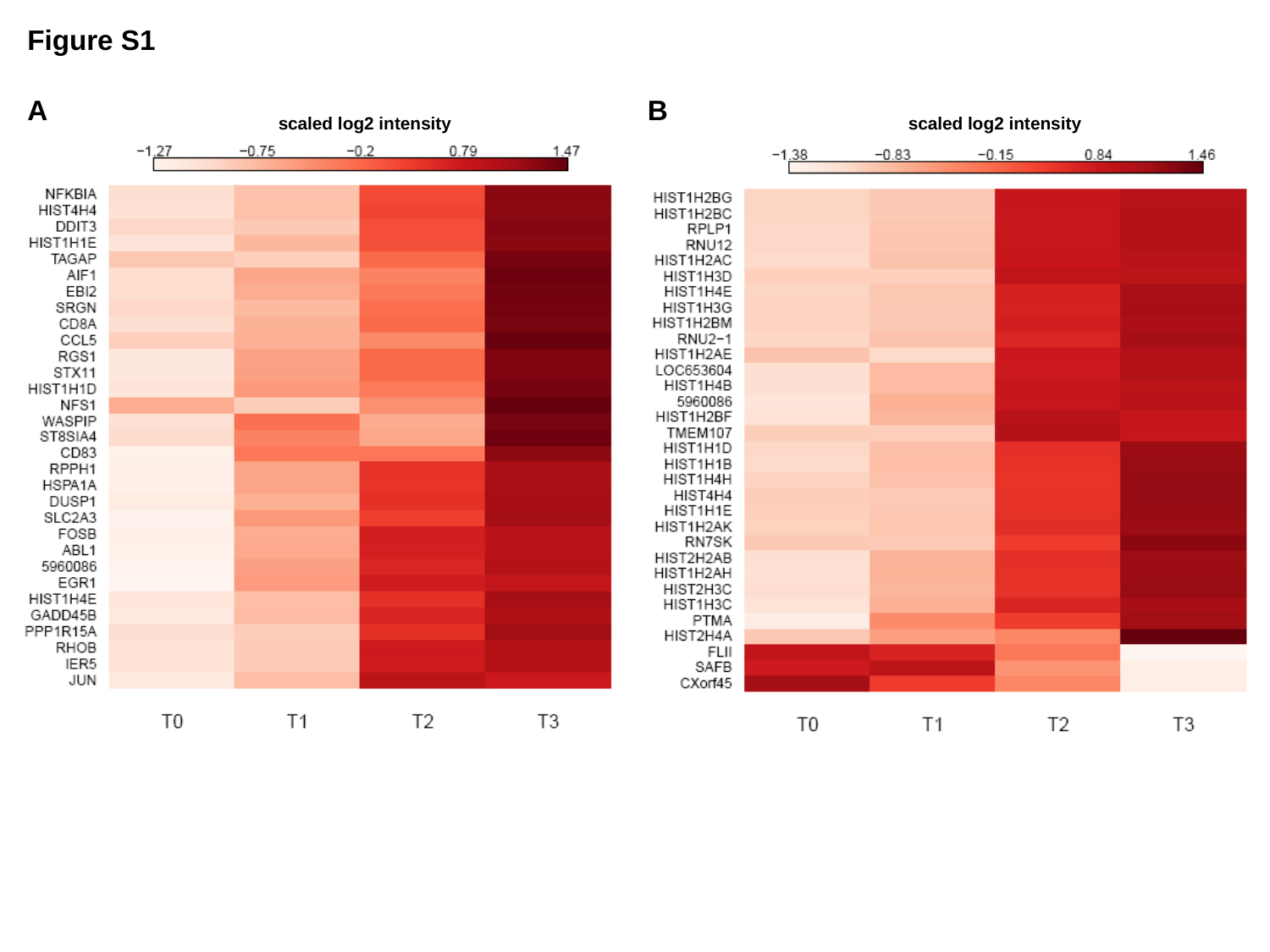

Figure S1
A
B
scaled log2 intensity
scaled log2 intensity

Supplement: Figure S1 — Expression alteration at each time point in the Tumor (A) and Normal (B) datasets (PPTX) [file pone.0053406.s001.pptx]

## Slide 1
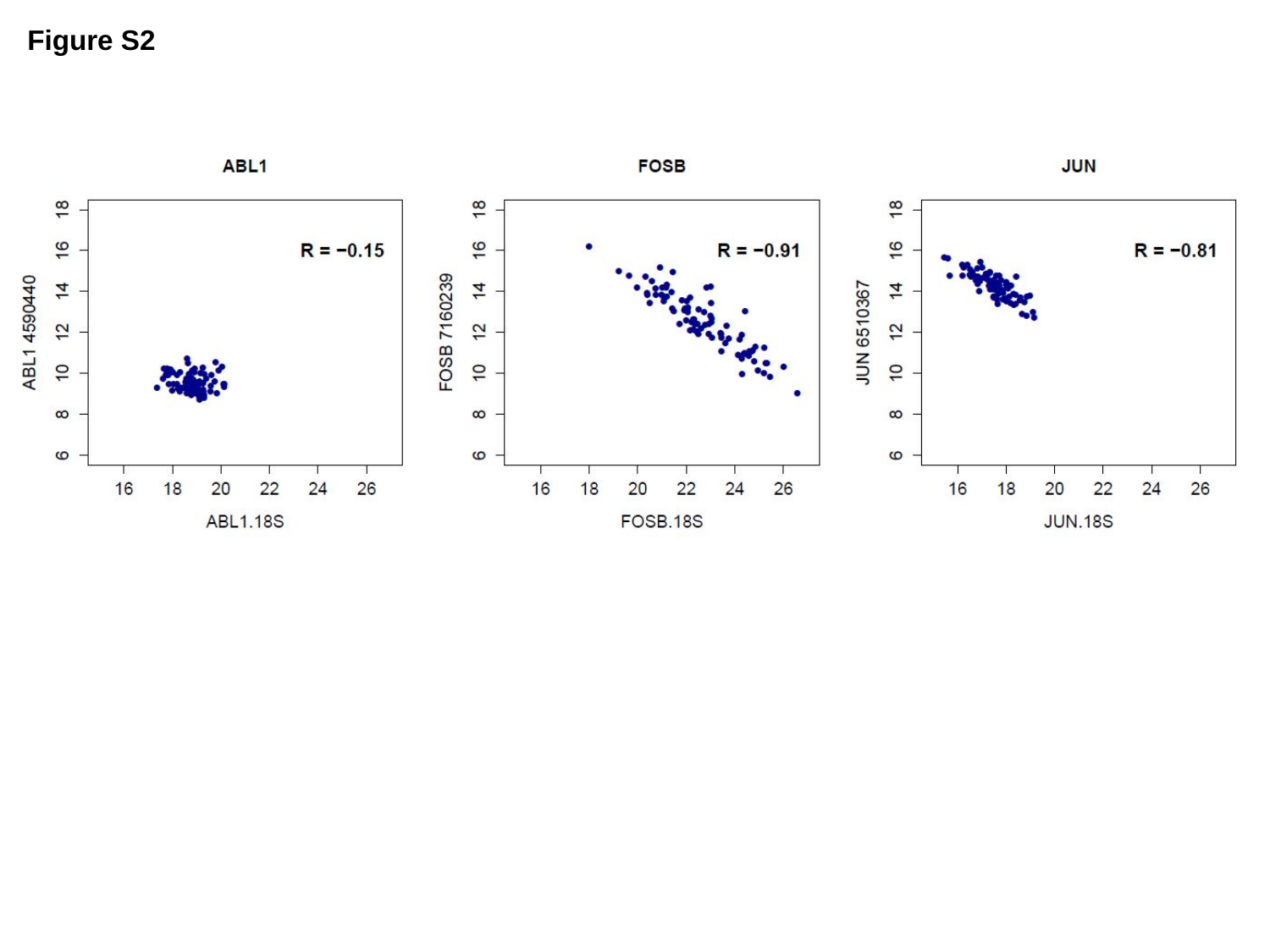

Figure S2

Supplement: Figure S2 — Scatter plots of RT-PCR ΔCt values (x-axis) versus log2 microarray intensity values(y-axes) for ABL1, JUN and FOSB genes. In each graph the Pearson correlation coefficient (R) was used to measure the strength of association. (PPTX) [file pone.0053406.s002.pptx]
